# The Drop That Spilled the Cup: Acute Myocardial Infarction in a Young Woman with Underlying Thrombophilic Polymorphisms and Oral Contraceptive Use

**DOI:** 10.1155/2014/249715

**Published:** 2014-12-23

**Authors:** Nunzio Russo, Enrico Franzì, Gianfranco Capilli, Anella Antonietta Patané, Silvia Paola Russo, Rosario Evola

**Affiliations:** ^1^Unit of Cardiovascular Disease, Hospital San Vincenzo, Contrada Sirina, 98039 Taormina, Italy; ^2^Cardiac Intensive Care Unit, Hospital San Vincenzo, Contrada Sirina, 98039 Taormina, Italy; ^3^Cardiovascular Imaging, Hospital San Vincenzo, Contrada Sirina, 98039 Taormina, Italy; ^4^Cardiac Catheterization Lab, Hospital San Vincenzo, Contrada Sirina, 98039 Taormina, Italy; ^5^Vita-Salute San Raffaele University, San Raffaele Hospital, 58 Via Olgettina, 20132 Milan, Italy

## Abstract

We present the case of a 28-year-old woman who was admitted to our cardiology unit for acute coronary syndrome. Her history was notable for cardiovascular disease familiarity, active smoking, and oral contraceptive use. On further analysis, she was noted to have thrombophilic polymorphisms involving the plasminogen activator inhibitor (*PAI*), angiotensin-converting enzyme (*ACE*), and methylenetetrahydrofolate reductase (*MTHFR*) genes. We discuss the implications that these cofactors may have had in the genesis of the disease.

## 1. Case Description

A 28-year-old woman was admitted for acute coronary syndrome (ACS).

She was born at term naturally to nonconsanguineous parents. Menarche was at age 13. She had a 3.5 pack-year smoking history, had regularly been smoking marijuana for some years, and had been taking combined oral contraceptives (COCs) for 10 years. Specifically, she was treated with ethinyl estradiol and gestodene until 2012; after that she was switched to new-generation COC ethinyl estradiol and drospirenone, which she took until admission. She had two at-term pregnancies at the age of 25 and 26. The first of these was characterized by gravid hypertension and the other by hyperfibrinogenemia, which was treated with low-molecular-weight heparin (LMWH). Her family history was remarkable for early-onset paternal ischemic heart disease (IHD): her father was affected since age 45 and a paternal uncle had an acute myocardial infarction (AMI) which required coronary artery bypass graft (CABG) at age 42.

**Figure 1:**
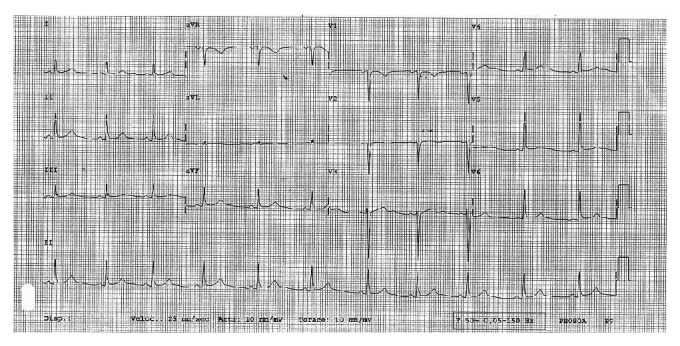
ECG performed 6 hours after presentation to the ED, when symptoms had regressed. The tracing shows lack of R-wave progression from V1 to V3 and is otherwise nondiagnostic.

**Figure 2:**
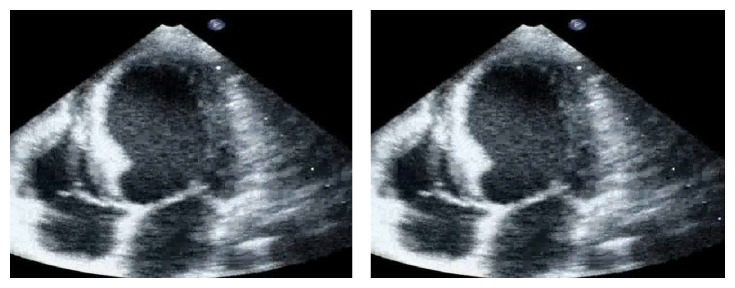
Transthoracic echocardiogram performed in the ED showed akinesia of left ventricular medium-distal apical septum and medium-distal anterior walls, with a 40% EF (see video).

**Figure 3:**
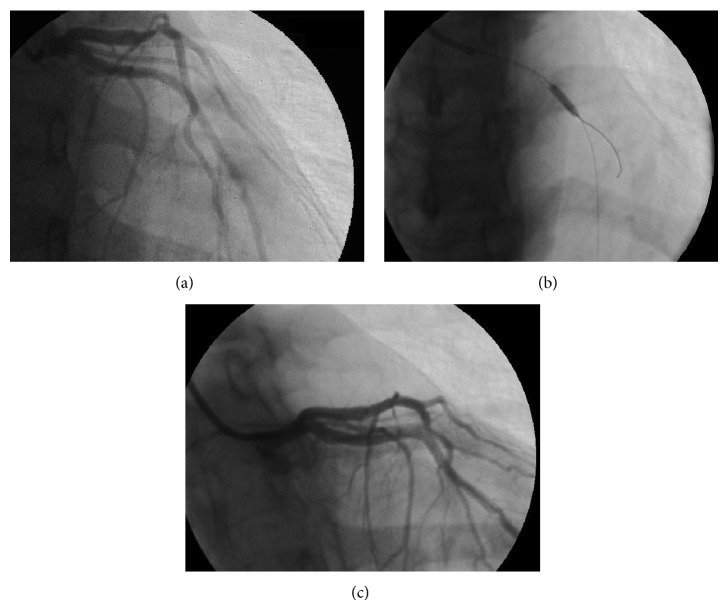
Cardiac catheterization study. (a) Marked stenosis of the anterior descending coronary artery can be seen in the upper left corner. (b) A catheter containing the deflated balloon is advanced through the restriction. (c) After balloon angioplasty and drug-eluting stent placement, contrast dye is injected again to verify complete dilatation of the stenosis.

**Figure 4:**
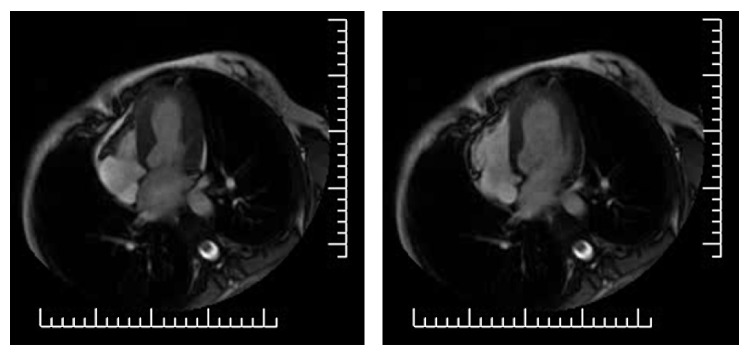
Contrast-enhanced CMR imaging performed 1 month after discharge shows normal ejection fraction and contractility, as well as viable, nonfibrotic myocardium.

Three days before admission, moderate exertion caused left arm pain, which superseded with cessation of activity. On the day of admission, at 8.30 a.m., the patient reported precordial chest pain with a pressure quality, presenting at rest and regressing spontaneously. The same symptoms recurred after some minutes, with higher intensity and accompanied by diaphoresis. The patient was therefore brought to the local emergency department (ED). When she arrived, she was symptom-free. Physical examination was unremarkable. An electrocardiogram (ECG) was performed six hours after presentation, while the patient was still symptom-free, and resulted to be within limits (Figure 1). Cardiac necrosis biomarkers were measured on serum specimens: a small increment of creatine kinase MB (CK-MB) and of troponin I was noted (Table 1). Cardiac consultation was hence requested. Transthoracic echocardiography (Figure 2 and video (in Supplementary Material available online at http://dx.doi.org/10.1155/2014/249715)) showed akinesia of left ventricular medium-distal apical septum and medium-distal anterior walls, with a 40% ejection fraction (EF). The patient was therefore admitted to the cardiac catheterization laboratory for coronary artery catheterization. While the exam was being arranged, the patient suffered a cardiac arrest due to ventricular fibrillation, which was promptly cardioverted to sinus rhythm with direct current (DC) defibrillation. Coronary artery catheterization revealed subocclusive calcific stenosis of the proximal tract of the left anterior descending coronary artery (Figure 3), which was treated with heparin, percutaneous angioplasty (PTCA), and the implant of a drug-eluting stent (DES), followed by administration of 200 mg aspirin and 300 mg clopidogrel loading dose. The patient was then started on a daily regimen of 100 mg aspirin and 75 mg clopidogrel. Five days after admission, cardiac magnetic resonance (CMR) imaging evidenced recovery of apical and interventricular septum contractility with normal left ventricular function and nonfibrotic, viable myocardium (Figure 4).

Thrombophilia was suspected in this patient. Antinuclear antibodies (ANA) had low-grade positivity (1 : 80), and antiphospholipid (APL) and anti-double-stranded DNA antibodies were however negative. Modest positivity (1 : 80) was also noted for anti-smooth muscle antibodies (ASMA). V Leiden factor was absent and proteins C and S had a normal activity. The patient was homozygous for the methylenetetrahydrofolate reductase (*MTHFR*) polymorphism A1298C and heterozygous for the* MTHFR* C677T polymorphism. Serum homocysteine levels were, however, within normal limits. Antithrombin III activity was not measured due to ongoing therapy with unfractionated heparin and, subsequently, low-molecular weight heparin.

On further genetic testing, homozygous polymorphism 4G/4G was present on the plasminogen activator inhibitor (*PAI*) gene (normal variant 5G/5G), as well as the D/D polymorphism on the angiotensin-converting enzyme (*ACE*) gene.

The patient was discharged with double antiplatelet therapy (DAPT), a statin, a beta-blocker, and an ACE inhibitor. At 30-day follow-up, the patient was asymptomatic and maintained a stable hemodynamic status.

## 2. Discussion

Cardiovascular diseases represent the main cause of death in western countries and as such are an important social and economic problem. Classical risk factors for arterial thrombosis (e.g., diabetes, hypertension, and hypercholesterolemia) are much less prevalent in young people than they are in the elderly population. The World Health Organization (WHO) has outlined the increasing exposure of younger age groups to emerging risk factors: women on COC are fivefold more likely to develop AMI [1] and a procoagulant state secondary to autoimmune diseases and genetic polymorphisms than nonexposed age-matched populations. The reason behind such association lies within the many interactions that exist between COCs and the hemostatic system: ethinyl estradiol can indeed induce resistance to activated protein C and increased synthesis of factors II, V, and VII [2]. The end-result is a prothrombotic state, which even appears to be potentiated in thrombophilic conditions.

The first case report of AMI secondary to COC use was published by Boyce et al. in 1963 [3]. The risk of arterial thrombosis in relation to oral contraceptives (RATIO) trial [4] went on to identify various arterial thrombosis risk factors in young women. In particular, high PAI1 levels and hypofibrinolysis were associated with an increased rate of AMI in young women treated with COC. Moreover, the PAI 4G/4G polymorphism, which this patient inherited, has been associated with a higher risk of coronary artery disease (CAD) [5]. The D/D ACE genotype is associated with high circulating levels of angiotensin II and is particularly represented in AMI patients [6]. This patient had pregnancy-related hypertension:* ACE* D/D homozygosity can potentially contribute to the pathogenesis of essential hypertension [7], as well hypertension at a young age and low body weight at birth [8]. These two genotypes code for the regulation of fibrinolysis in response to vascular lesions after angioplasty and are associated with a high hazard of restenosis after PTCA [9].

The patient was also a carrier of MTHFR gene polymorphisms associated with CAD, peripheral artery disease (PAD), stroke, and neural tube defects [10].

Finally, numerous studies point to the exponential increase of AMI risk with the concomitant use of COC and cigarette smoking, especially in women older than 35 years [11, 12]. Recently, Lidegaard et al. have observed that women who smoke and take COC have a relative risk (RR) for stroke and AMI of 1.57 (95% confidence intervals, CI 1.31–1.87) and 3.62 (95% CI 2.69–4.87), respectively [13]. However, Tanis et al. have failed to confirm this interaction with the newer, third-generation COCs, which this patient took [14].

## 3. Concluding Remarks

COC may have played a critical role in the pathogenesis of AMI in this patient, being the “drop that spilled the cup” in a premorbid condition characterized by smoking habit and genetic predisposition, thus leading to the perfect coronary thrombotic storm.

## Supplementary Material

Transthoracic Echocardiogram: an apical four-chamber view demonstrates akinesia of left ventricular medium-distal apical septum and medium-distal anterior walls, with a 40% ejection fraction.

## Figures and Tables

**Table 1:** Serum cardiac biomarkers.

Biomarker	Measured value	Reference range
CK	290 IU/L	30–135 IU/L
CK-MB	33 IU/L	Less than 6% of measured CK
Troponin I	6.75 ng/mL	Less than 0.40 ng/mL

IU: international units; CK: creatine kinase; CK-MB: muscle-brain type creatine kinase.
